# Acetyl-cholinesterase-inhibitors reconsidered. A narrative review of post-marketing studies on Alzheimer’s disease

**DOI:** 10.1007/s40520-023-02675-6

**Published:** 2024-02-07

**Authors:** Giovanni Zuliani, Marco Zuin, Tommaso Romagnoli, Michele Polastri, Carlo Cervellati, Gloria Brombo

**Affiliations:** Department of Translational Medicine and for Romagna, University of Ferrara, Ferrara, Italy

**Keywords:** Alzheimer’s disease, Acetyl-cholinesterase-inhibitors, Cognitive decline, Dementia, Mortality

## Abstract

The real efficacy of Acetyl-cholinesterase-inhibitors (AChEI) has been questioned. In this narrative review we evaluated their effect on cognitive decline, measured by Mini Mental State Examination (MMSE), and on total mortality rates in patients with Alzheimer’s disease (AD) recruited into post-marketing open/non-randomized/retrospective studies. In AD patients treated with AChEI, the mean MMSE loss ranged from 0.2 to 1.37 points/years, compared with 1.07–3.4 points/years in non-treated patients. Six studies also reported data about survival; a reduction in total mortality relative risk between 27% and 42% was observed, over a period of 2–8 years. The type of studies and the use of MMSE to assess cognitive decline, may have introduced several biases. However, the clinical effects of AChEI seem to be of the same order of magnitude as the drugs currently used in most common chronic disorders, as regards progression of the disease and total mortality. In the absence of long-term randomized trials on “standard” unselected AD outpatients, open/retrospective studies and health databases represent the best available evidence on the possible effect of AChEI in the real-word setting. Our data support the clinical benefit of AChEI in older patients affected by AD.

## Introduction

Dementia is a one of the most important health problems in the older populations; the number of patients is approximately 55 million worldwide, with 10 million new cases per year [1, 2].

Alzheimer’s disease (AD) represents the most common cause of dementia worldwide, accounting for at least two-thirds of cases of dementia in people age 65 and older [1, 3–5].

Although the research of new effective therapies for AD has never stopped, no new drugs have been approved in the last two decades, except for the two “debated” monoclonal antibodies Aducanumab and Lecanemab [6, 7]. Of consequence, Acetyl-cholinesterase (AChE) inhibitors (AChEI), including Donepezil (DON), Galantamine (GAL), and Rivastigmine (RIV) still represent the main pharmacological treatment in AD patients. With the aim of ameliorating the symptoms and slowing the progression of the disease [8, 9]. DON is a reversible inhibitor of AChE with a very long half-life. RIV is a pseudo-irreversible inhibitor of AChE and of Butyrylcholinesterase too, with a very short half-life, while GAL is a reversible inhibitor of AChE, and a presynaptic modulator of the nicotinic receptor with a short half-life. These three drugs are indicated in patients with mild to moderate AD, i.e. with a Mini Mental State Examination (MMSE) score between 10 and 26/30.

The clinical efficacy of AChEI has been demonstrated by phase-3 randomized double-blind controlled trials (RCTs) in patients with mild-moderate AD, and a follow-up more often shorter than 1 year. However, the efficacy of AChEI in the real world setting has been questioned. In one hand, the results of the RCTs (“the gold standard”) are distant from the clinical practice, since they enrolled strictly-selected patients that do not represent the typical outpatient’s population. On the other hand, it is difficult to compare the progression of dementia in patients treated with AChEI with the “hypothetical progression” in the same patients if not treated. Actually, some observational/open/non-randomized/retrospective trials have reported data about the progression of AD in both patients treated and not treated with AChEI.

The aim of the present narrative review is to summarize the results of available studies on AChEI in AD patients from the “real-world” setting, with the aim providing an approximate estimate of the real clinical efficacy of these drugs.

## The phase-3 RCTs with AChEI in Alzheimer’s disease

In recent years, three large meta-analyses have analysed the AChEI efficacy in AD.

The first, published by Kobayashi et al. in 2015, based on a Bayesian network meta-analysis, evaluate the safety and efficacy of AChEIs in patients with mild-to-moderate AD [10]. Their analysis included 21 studies with AChEI enrolling 9509 AD patients, with a mean age from 69 to 78 years. The mean length of follow-up ranged from 12 weeks and 1 year, while the average MMSE score ranged from 15.1 to 21.5/30 (moderate dementia). All treatments with DON, RIV or GAL at different dosages, were significantly more efficacious than placebo in cognition (measured by ADAS-Cog) while all treatments, except GAL, were significantly more efficacious than placebo in global change in the Clinician’s Interview-Based Impression of Change Plus caregiver input (CIBIC-Plus) or Clinical Global Impression of Change (CGIC). However, no efficacy was observed in neuropsychiatric symptoms. Furthermore, this network meta-analysis revealed that all drugs had a significantly greater improvement than placebo; the derived hierarchy was GAL > RIV > DON for cognition, and DON > RIV > GAL for global change. The authors concluded that the results indicated benefits in cognition and clinical global change, but the efficacy on neuropsychiatric symptoms was questionable.

In 2018 Knight et al. published a systematic review and meta-analysis of the effectiveness of AChEI (and memantine) in treating the cognitive symptoms of dementia [11]. Overall, the analysis included 55 studies on AD, 9 studies on vascular dementia (VD), 4 studies on mixed AD/VD dementia, 10 studies on Lewy Body disease (LBD)/Parkinson’s dementia (PDD), and 2 studies on Fronto-Temporal dementias. The average age of patients was 73.8 years, while the follow-up ranged from 4 week to 2 years. The average MMSE score was 18.6/30 (moderate dementia). Forty studies investigated DON, 13 investigated GAL, and 13 investigated RIV. Meta-regressions of the AChEI results at 3 and 6 months identified only the dementia type as a possible moderator of treatment effect. Treatment effects were smaller for patients with AD or VD (0.97 MMSE points at 3 months; 0.91 points at 6 months) than for LBD/PDD dementias (1.99 MMSE points at 3 months; and 2.11 points at 6 months). The effects after 1 year were reported only for AD and VD, and indicated an effect similar to those at 3 and 6 months (1.10 points). The authors concluded that AChEI demonstrate a modest effect compared to controls on MMSE score, which is moderated by dementia sub-type.

Once again in 2018, Dou et al. published a network meta-analysis comparing the safety and effectiveness of AChEI (and memantine) for AD [12]. The analysis included 41 studies of which 32 only with AChEI, for a total number of over 18,000 patients. The mean age ranged from 70 to 84 years, while the mean length of follow-up was 29 weeks (from 1 week to 2 years). The MMSE score ranged from 8 to 24/30 (severe-moderate-mild dementia). As regards cognitive functions, in mild-moderate AD all drugs (DON, GAL, and RIV), except for RIV 5-cm^2^ patch, were significantly more efficacious than placebo, with a probability to be the best choice to enhance cognitive performance GAL 32 mg > GAL 24 mg > DON 10 mg > RIV 15-cm^2^ patch. In terms of secondary outcomes, 20 studies reported data on the activities of daily living according to ADLs and BADLs scale. All drugs, except for RIV 5-cm^2^ patch, were significantly more efficacious than placebo. RIV 15-cm^2^ patch was likely to be the best treatment to improve activities of daily living. The analysis indicated that no interventions had significant improvements in neuropsychiatric symptoms compared with placebo. The assessment of clinical global changes with 22 eligible studies and 11 treatments on the CIBIC + scale and the CGIC scale indicated that DON (5 mg, 10 mg, 23 mg) as well as RIV (12 mg, 10-cm^2^ patch, and 15-cm^2^ patch) were significantly more efficacious than placebo. The authors concluded that the pharmacological interventions with AChEI have beneficial effects on cognition, function, and global changes, but not on neuropsychiatric symptoms.

Overall, what emerges from these meta-analyses is:

1. The RCTs that evaluated the efficacy of AChEI have a short follow-up (more often < 1 year) compared with the average length of AD (8 years after symptoms appear); this aspect prevents the possibility of understanding the real effect of AChEI on the evolution of the disease.
2. Most of AD patients had, at enrolment, a MMSE abundantly lower than 24/30, which means that the disease was not at its onset (moderate to severe). This aspect does not allow a direct comparison with monoclonal antibodies, which have been recently tested in MCI/early AD.
3. The clinical effect of AChEI was “mild”, but it was significant when compared with placebo as regards cognition, global change, and function.

## The results of the studies from the “real world”

After the publication of the phase-3 RCTs on AChEI, some studies were published regarding the efficacy of these drugs in the clinical practice. Most of available data came from open/non-randomized/retrospective studies or public health databases reporting consecutive evaluations of the cognitive status (by MMSE) in AD outpatients treated or not treated with AChEI, as well as a couple of post-marketing RCTs. This type of studies have several well-known limitations; however, they represent the only possibility in trying to understand the “true” efficacy of AChEI after placing on marketing.

First, it is worth to cite the study of Mendiondo et al. [13]. By using the Consortium to Establish a Registry for Alzheimer’s Disease (CERAD) database, these authors modelled the MMSE change as a function of time. MMSE is widely used in memory clinics to measure dementia severity in AD. The rates of progression calculated by Mendiondo ranged between 1.4 and 1.8 points/year for MMSE score between 20 and 24/30, 2.7–4.2 points/years for MMSE between 15 and 19/30, and 3.3–5.5 points/years for MMSE between 10 and 14/30. Age significantly affected the curve of MMSE decline, while education and sex did not. Heterogeneity in the rate of change was observed, due to inter-rater comparisons, environmental and patient factors (day-to-day fluctuations and disease heterogeneity).

### Studies reporting cognitive decline and/or mortality rates in patients treated with AChEI

In 2005 Small et al. evaluated data from two pooled open-label extensions of four 6-month, randomised, placebo-controlled trials with RIV [14]. The mean baseline MMSE score was 19.3/30. As reported by Bullock and Dengiz [15], in 1998 patients treated with RIV, the loss in MMSE score was 5 points over 5 years (1.2 point per year), compared with 12 points MMSE (2.4 point per year) as predicted by the model for untreated patients. They concluded that, after an initial period of stabilization, a cognitive decline will follow also on AChEI treatment, but it would be slower and later than expected if the patients were left untreated.

In 2004 Bellelli et al. presented the data from the CRONOS project in the Lombardia region of Italy [16]. The authors reported the results of a 36 weeks (9 months) follow-up in a sample of 808 patients with AD treated with AChEI; of them, 441 were *naïve*, (had never used AChEI before) while 368 were not *naïve*. After 12 weeks, both groups improved their MMSE score (+ 0.8 and + 0.5 points, respectively). After 9 months, the average MMSE loss was 0.3 points. In detail, the MMSE score was + 0.1 points in *naïve* patients, and − 1.2 points in non-*naives*.

In 2007 Wallin et al. reported longitudinal data from 435 outpatients with diagnosis of AD treated with DON, enrolled into the Swedish Alzheimer Treatment Study (SATS) [17]. After 3 years of treatment, the mean MMSE score change from baseline − 3.8 points (− 1.27 points/year). As noted by the authors, the mean MMSE score change from baseline was positive for more than 6 months, and in a subgroup of patients also for 12 months.

In 2009 Nelson et al. evaluated the association between AChEI therapy and the rate of cognitive decline in a sample of 65 patients with AD (n.45) or AD + LBD (n.20) [18]. The MMSE was > 20/30. They employed a nonlinear regression model to correlate the rate of pre-mortem cognitive decline (MMSE) with different groups based on neuropathological diagnosis. The covariates considered were treatment with AChEI, sex, educational level, Apo E genotype, and pathology (pure AD vs AD/LBD). Untreated AD patients lose half their maximum MMSE score approximately 1.4–1.7 years earlier compared to treated AD patients (depending on APO E status). Among patients not bearing APO E4 allele, those treated with AChEI lose, on average, 1.2 points MMSE per year, compared with 1.6 point of those not treated, with a benefit of more than 4 points in 12 years; the curves describing MMSE decline over time started to diverge after 3 years. The authors concluded that patients with dementia taking AChEI showed a MMSE scores that decline at a slower rate than patients not taking the drugs.

In the same year, Calabria et al. investigated the long-term efficacy of the ACHEI treatment in 427 patients with mild-to-moderate AD, followed for a period of 21-months [19]. At the end of the study, first-time drug takers (“Naives”: mean age: 78.5 years; mean MMSE: 18.6/30) declined by 1.2 MMSE points (0.69 points/year). Predictors of responsiveness were MMSE score at baseline and at 3 months of treatment.

In 2011 Wattmo et al. analysed the data from the SATS, an open, 3-year, non-randomized prospective study in which AD patients were treated with AChEI in a routine clinical setting [20]. Patients were assessed at baseline and every 6 months, using several rating scales including MMSE. This study enrolled 843 patients with a mean age of 75 years; the MMSE ranged from 10 to 26/30. The MMSE mean difference from the baseline score (95%CI) was − 0.6 (− 0.8 to − 0.3) after 1 year of AChEI treatment, − 2.3 (− 2.7 to − 1.9) after 2 years, and − 3.2 (− 3.7 to − 2.7) after 3 years. Approximately, AD patients treated with AChEI lost 1 point MMSE per year. Higher mean dose of AChEI, male sex, and older age were some predictors of better short-term response.

In the same year, Kavanagh et al. evaluated the long-term effects of GAL on MMSE score in 258 AD patients treated for up to 5 years, using both clinical data and epidemiological modeling [21]. The MMSE ranged from 7 to 25/30. Re-contacted study investigators obtained data from AD patients originally recruited into three RCTs involving GAL. In the absence of long-term placebo, the rate of cognitive decline without treatment was projected using the model of Mendiondo [13]. On average, patients treated with GAL lose 5 points MMSE in 5 years (1 point per year) compared with 15 point as expected in not-treated patients (3 points per year). In patients with MMSE 22/30 treated with GAL the gain was about 5 points MMSE in 5 years, while in patients with MMSE 16/30 it was about 4 point in 5 years.

Again in 2011, Wallin et al. explored the three-year effects of GAL treatment in a sample of 280 patients (62% females) with AD, enrolled in the SATS [22]. The mean age was 73.2 years, while the average MMSE score at baseline was 23.3/30. After 3 years of treatment, the mean change in MMSE score from baseline was 2.6 points (0.87 point/year). Globally, half of the patients improved or remained unchanged for at least 2 years.

In 2013 Nordstrom et al. evaluated over 7000 older subjects with AD or mixed dementia (AD + VD) (mean age: 79 years) from the Swedish Dementia Registry (SveDem) [23]. After propensity score matching, 1676 patients treated with AChEI were compared with 1676 patients not treated. The average follow-up length was 495 days. AChEI use was associated with a lower risk of overall death (HR: 0.65; 95%CI: 0.54–0.76) and myocardial infarction (MI) (HR: 0.62; 95%CI:0.40–0.95). Patients taking the highest AChEI doses (DON 10 mg, RIV > 6 mg, GAL 24 mg) had the lowest risk of death (HR:0.54, 95%CI:0.43–0.67) or MI (HR:0.35, 95%CI:0.19–0.64) compared with those who had never used AChEI.

In 2014 Hager et al. evaluated the effect of GAL in a multicenter RCT by comparing 1025 patients treated with GAL versus 1021 patient on placebo [24]. The mean age was about 73 years, while the follow-up length was 2 years. The average MMSE score was 19/30. MMSE score significantly worsened in the placebo group (− 2.14 points; 1.07/year) compared with the GAL group (− 1.41; 0.70/year). The mortality rate was significantly lower in the GAL group versus placebo (HR: 0.58; 95%CI: 0.37; 0.89). The authors concluded that, in mild to moderate AD patients, the long-term treatment with GAL significantly reduced mortality and the decline in cognition.

In 2015 Wattmo et al. examined the possible association between long-term AChEI therapy and survival by analyzing data from the SATS [25]. The study included 1021 patients with diagnosis of AD; the baseline MMSE ranged to 10 to 26/30. After up to 16 years of follow-up, 841 (82%) of the participants had died. In Cox multivariate models, higher AChEI dose was an independent predictor of increased life expectancy. The frequency of death over the first 3 years was lower among individuals who received a higher AChEI dose compared with a lower dose (9 vs 18%, p < 0.001), regardless of drug agent. After up to 16 years of follow-up, the corresponding percentages were 79 versus 86% (p: 0.005). Individuals who received a higher dose of AChEI had a longer survival than those who received a lower dose (6.4 ± 2.9 years vs 5.5 ± 2.8 years, p < 0.001). Besides, a longer time of exposure to AChEI was an independent predictor of increased life expectancy in the multivariate models. The authors concluded that a longer survival time can be anticipated for AD patients with slower deterioration who receive and tolerate higher AChEI doses and a longer duration of treatment.

In 2015, Nakano et al. retrospectively analyzed the long-term efficacy of GAL treatment in 279 very elderly AD patients from the Okayama Galantamine Study [26]; the patients were evaluated at baseline and after 3, 6, 12, and 24 months. The mean age was 80.6 years, 60.9% were females. The number of patients evaluated with the MMSE was 268 (mean score at baseline: 20.0/30). After 12 months, the average decrease in MMSE score reported was 0.20 points, while after 6 months an average improvement of 0.5 MMSE points was described. A better efficacy of GAL was noted for male sex, and baseline lower cognitive, affective, and ADL functions.

In 2017, Nakagawa et al. evaluated the long-term efficacy of GAL for AD in routine clinical practice by conducting a 72-week post-marketing surveillance study [27]. To this aim, 661 patients with mild-to-moderate AD were enrolled; of them 554 were assessed for efficacy. The mean age was 78.9 years, while the mean MMSE score was 18.9/30. The mean change from baseline to 72 weeks was − 1.21 MMSE points (0.88 points/year).

In 2018 Mueller et al. investigated the possible association between AChEI and mortality by using a large mental healthcare database in South London, linked to Hospital Episode Statistics and Office for National Statistics mortality data, to assemble a retrospective cohort [28]. The study included 2464 AD patients, of which 1267 treated with AChEI. The mean age was 82 years, while the average MMSE score was 19/30. The mean length of follow-up was 3.7 years. After a 1:1 propensity score matching, 953 receiving AChEI were compared to 953 not-treated. Mean MMSE score was 19.8/30, while the mean follow-up time was 3 years. Multivariate Cox regression model showed a strong association between AChEI prescription and reduced all-cause mortality, that remained significant after adjusting for a large number of covariates (H.R:0.77; 95%C.I.:0.67–0.87). The results suggest that people with AD who are prescribed AChEI have reduced mortality by approximately 40%, half of which remained independent of a range of potential confounding factors. The authors concluded that as AChEIs are now established in clinical practice and well-tolerated, depriving patients of AChEI prescription would be considered unethical.

In 2020, Vaci et al. investigated the real-world effectiveness of AChEI (and memantine) for dementia-causing diseases in a large UK observational secondary care service [29]. The sample included 7415 individuals with AD, patients with mixed dementia and VD. On average, after 4 years of follow-up, patients with mild dementia lost 5.3 MMSE points (1.37 points/year), while patients with moderate dementia lost 3 MMSE points (0.75 points/year). The authors underlined the fact that 68% of individuals responded to treatment with a period of cognitive stabilization before continuing their decline at the pre-treatment rate.

More recently Xu et al. published a study based on the SveDem [30]. The sample included 31,054 patients with AD of which 21,826 treated with AChEI. After propensity score matching, 11,652 patients treated with AChEI were compared with 5826 non-users. The mean age of the sample was 81.2 years, while the average MMSE score was 21.2/30. The mean length of follow-up was 5 years. The average yearly reduction in MMSE score was − 1.62 (95%CI: − 1.70 to − 1.54) points in the whole sample. AChEI use was associated with higher MMSE at each visit (0.13 MMSE points/year), and with a 27% lower risk of death (H.R.: 0.73; 95%CI: 0.69–0.77) compared with non-users. Patients who took higher dose of AChEI had lower mortality risk in a dose-dependent response. GAL was associated with a lower risk of death (H.R.: 0.71; 95%CI:0.65–0.76). It was concluded that AChEI are associated with modest cognitive benefits that are persisting on long term; moreover, they are associated with reduced mortality risk, which may be partly explained by the cognitive effects.

In 2022, Zuin et al. evaluated the effect of AChEI on cognitive decline and overall mortality in a sample of older patients with AD, VD or LBD enrolled between 2005 and 2020 by the National Alzheimer’s Coordinating Center Uniform Data Set [31]. Patients included into the study met the following criteria: (1) age ≥ 65 years; (2) diagnosis of dementia; (3) MMSE score ≥ 10/30 (mild-moderate dementia); (4) had at least 2 in-person visits, with 2 years of follow-up; and (5) were not receiving memantine. The mean follow-up period was 7.9 years. A 1:1 propensity score matching was performed generating a cohort of 1.572 patients (786 treated with AChEI and 786 not treated). Focusing on AD (625 vs 616 subjects), MMSE score declined in the two groups over time, and the curves started to diverge after about 3 years. On average, at the end of follow-up patients not treated with AChEI displayed a greater reduction compared with those treated (MMSEΔ: − 10.8 vs -5.7 points) (*p* < 0.001). In the adjusted model, AD patients using AChEI had an estimated average gain of + 0.88 points MMSE per year than patients not treated. At multivariate Cox regression analysis (adjusted for age, gender, dependency level, depression and baseline MMSE) a strong association between AChEI therapy and lower all-cause mortality in AD patients was found (H.R.: 0.67; 95%CI: 0.52–0.82). Although the study had several limitations, the authors concluded that, among older people with mild AD, treatment with AChEI was associated with a slower cognitive decline and with reduced mortality rates.

#### Other reports on this topic

In 2005, Zurlo et al. reported the data on 437 older AD patients (mean age 78.8 years) enrolled by the Geriatric Memory Clinic of Ferrara, Italy [32]. The mean MMSE score at entry was 20.3/30, while the follow-up was up to 51 months. Patients treated with AChEI lost, on average, 0.95 point MMSE per year, compared with 2.6 point per year in patients not treated.

In the same year, Bonati et al. reported their data on AD older patients enrolled by the Geriatric Memory Clinic of Reggio-Emilia, Italy [33], with a follow-up of 56 months. Subjects treated with AChEI lost, on average, 0.96 points MMSE per year, while patients not-treated with AChEI lost, on average, 2.9 point MMSE per year.

In Table 1 are summarized the average annual loss in MMSE score reported in different studies on AD patients treated with AChEI; the MMSE loss ranged from 0.2 to 1.37 points/years, with a projection to 4 years from 0.80 to 5.5 points, assuming a constant rate of decline.

**Table 1:** Mean annual loss in Mini Mental State Examination (MMSE) score in patients with Alzheimer’s Disease (AD), treated with acetyl-cholinesterase-inhibitors (AChEI)

Author	Sample size (n.)	Mean age (years)	Baseline MMSE score	Follow-up (years)	Mean MMSE loss (points/year)	4 Years projection
Vaci [29]^a^	7415	Adults	21/30	4 years	Mild Dem: 1.37	5.5
Wallin [17]	435	74.6	22	3 years	1.26	5.04
Nelson [18]^b^	33	76.6	21/30	12 years	1.2	4.8
Small [14]	1998	–	19/30	5 years	1.2	4.8
Kavanagh [21]	258	73.3	–	5 years	1.0	4.0
Wattmo [25]	843	75	21.4	16 years	1.0	4.0
Zuin [31]	625	75	27/30	7.9 years	1.0	4.0
Bonati [33]	88	> 65	–	4.6 years	0.96	3.84
Zurlo [32]	126	> 65	21/30	4.2 years	0.95	3.8
Nakagawa [27]	661	78.9	18.9/30	1.4 years	0.88	3.52
Wallin [22]	280	73.2	23.3/30	3 years	0.87	3.48
Hager [24]	1015	72	19/30	2 years	0.70	2.8
Vaci [29]^a^	7415	–	21/30	4 years	Moderate Dem: 0.75	3.0
Calabria [19]	427	78.5	18.6/30	1.7 years	0.69	2.76
Bellelli [16]	808	77.2	19.2/30	9 months	0.40	1.6
Nakano [26]	268	80.6	20/30	2 years	0.20	0.80

Values are reported in decreasing order

^a^AD (+ Mixed dementia + VAD)

^b^Apo E4 negative

In Table 2 are summarized the mortality rates reduction observed in patients with AD treated vs not treated with AChEI (Hazard Ratio, 95% Confidence Interval). Overall, the reduction in mortality risk was between 27% and 42%, in a period of follow-up ranging from 2 to 8 years. It must also mention the study by Wattmo et al. [25] that, although did not report the Hazard Ratio for mortality, found that AChEI death frequency was lower in patients assuming higher vs low AChEI dose (9% vs 18% at 3 years, 79% versus 86% at 16 years); moreover, subjects receiving higher AChEI dose had a longer survival compared to those receiving a lower dose (6.4 vs 5.5 years).

**Table 2:** Relative and absolute mortality risk reduction in patients with AD, treated vs not-treated with acetyl cholinesterase-inhibitors

Author	Mean follow-up (years)	Hazard ratio	95%C.I	Relative risk reduction (%)	Absolute risk reduction (%)
Nordstrom [23]	2	0.64	0.54–0.76	36	8.7
Hager [24]	2	0.58	0.37–0.89	42	2.3
Mueller [28]	3.7	0.77	0.67–0.87	23	11
Xu [30]	5	0.73	0.69–0.77	27	3.1
Zuin [31]	7.9	0.67	0.52–0.82	33	4.5

### Studies reporting MMSE decline in patients not-treated with AChEI

Some studies reporting the trajectories of MMSE over time in AD patients not treated with AChEI will be briefly described below. It must be emphasized that, while these patients certainly did not take the AChEI, they could still be treated with other drugs including vitamins, nutraceuticals, or herbal products.

In 1993, Corey-Bloom et al. evaluated the clinical features and the rate of cognitive decline in older patients with either possible/probable AD or mixed dementia (AD + VD) from nine California Alzheimer’s Disease Diagnostic and Treatment Centers [34]. The length of follow-up was 1 year. Mean baseline MMSE scores for possible AD (n. 279), probable AD (n. 928) and mixed dementia (n. 430) were 17.9, 13.9, and 15.4/30, respectively. On average, all groups declined by about 2.8 points in 1 year.

In 1996 Rasmusson reported data from 132 AD patients with MMSE > 10/30, and examined at 6-months interval for a period up to 7.5 years (mean 2.5 years) [35]. The mean age was 70.9 years, while the average MMSE score was 16.7/30. The reported MMSE change per visit (6 month) was − 1.49 in males (2.98 points per year) and − 1.42 in females (− 2.84 point per year).

Again in 1998, Aguero-Torres et al. evaluated an established population aged 75 years and older searching for prognostic factors [36]. The sample included 133 cases of AD, 52 of VD, and 38 other types of dementia. The follow-up clinical examinations were programmed after 3- and 7-year intervals. The average rate of cognitive decline in the 81 mild to moderate subjects who survived 3 years was 2.4 MMSE points per year.

In 1999 Clark et al. reported data from 372 older patients with mild-moderate probable AD from CERAD with 1 or more years of follow-up (mean length: 2.4 years) [37]. The mean age was 71 years. They found that the average MMSE annual decline was − 3.4 points; however, a wide variability in the individual rate of decline was noted.

In 2000, Han et al. estimated the annual rate of change on MMSE in AD by means of a Literature search articles published from January 1981 to November 1997 [38]. Of the 439 studies screened, 37 studies involving 3492 AD patients followed over an average of 2 years were included into the meta-analysis. The pooled estimate of annual rate of change in MMSE score was − 3.3 points (95%CI: 2.9–3.7), with a significant heterogeneity of estimates across the studies.

In 2001, Doody et al. reported data from 298 AD patients with the aim of predicting disease progression during follow-up, by using the initial decline prior first physician visit [39]. Subjects with MMSE < 5/30 were excluded. The mean initial MMSE score was 20/30, while the mean age was 70 years. The calculated progression rate both pre-diagnosis and during follow-up was − 3.0 points MMSE per year.

In 2004, Suh et al. measured the rates of decline in cognition and function in sample of 107 AD patients from Korea, with a follow-up of 1 year [40]. The average annual rates of decline in the MMSE score were 2.3 points per year. Neither gender, duration of formal education, nor duration of AD since onset were predictors of cognitive or functional decline.

Again in 2004, Holmes et al. reported the cognitive status of 339 community-based AD patients (151 from London, and 188 from Oxford) with the aim of investigating the possible effect of butyrylcholinesterase K gene variation [41]. The mean age was 82 and 75 years, respectively. The length of follow-up was 3 years. None of the subjects was prescribed AChEI. The median annual MMSE decline for the whole sample was 2.3 points.

In 2011, Tschanz et al. evaluated, in a population-based sample of 328 incident AD cases, the progression of impairment in cognition, function, and neuropsychiatric symptoms [42]. The mean age was 85.9 years, while the average MMSE score was 21.9/30. Over a mean follow-up of 3.8 years, the mean annual MMSE change was − 1.53 points. Females declined more rapidly than males, with an average additional decline of 2.9 points over 3 years, 3.8 points over 5 years, and 4.1 points over 7 years.

In 2019, Stanley et al. compared the rate of cognitive decline in patients with AD aged < 65 years (young-onset), 65–74 years (middle-onset), and ≥ 75 years (late-onset) [43]. The study used longitudinal data from the Essex Memory Clinic, and included 305 participants; 56 had young-onset AD, 73 had middle-onset AD, and 176 late onset AD. There was evidence of a difference in cognitive decline across the age groups with the young-onset AD declining 2.8 MMSE points, middle-onset 2.0 MMSE points, and late-onset 1.4 MMSE points per year.

In Table 3 are summarized the average annual loss in MMSE score reported in patients with AD not treated with AChEI; the loss ranged from 1.07 to 3.4 points/years, with a projection to 4 years from 4.28 to 13.6 points, assuming a constant rate of decline. Overall, data suggest that most of AD patients lose < 3 MMSE points per years, and this is in good agreement with the current definition of “usual” AD cognitive decliner [44].

**Table 3:** Average annual loss in MMSE score in patients with AD, not-treated with AChEI

Author	Sample size (n.)	Mean age (years)	Baseline MMSE	Follow-up (years)	Mean MMSE loss (points/year)	4 Years projection
Clark [37]	372	71	–	2.4	3.4	13.6
Han [38]	3492	72.5	18.4	2	3.3	13.2
Doody [39]	298	70	20/30	3	3.0	12.0
Rasmusson [35]	132	70.9	16.7/30	2.5	2.9	11.6
Bonati [33]	88	> 65	–	4.6	2.9	11.6
Corey-Bloom [34]	1207	–	14.9	1	2.8	11.2
Mendiondo model MMSE 19/30 [13]	719	71.9	19/30	2	2.77	11.08
Zurlo [32]	126	> 65	21/30	4.2	2.6	10.4
Aguero-Torres [36]	133	> 75	–	3	2.4	9.6
Holmes [41]	339	79	18/30	3	2.3	9.2
Suh [40]	107	79.5	11.2/30	1	2.3	9.2
Stanley [43]	73	71.1	24.2/30	> 1	65–74 years: 2.0	8.0
Mendiondo model MMSE 21/30 [13]	719	71.9	21/30	2	1.77	7.08
Nelson [18]	12	76.6	22/30	12	1.6	6.4
Tschanz [42]	328	85.9	21.9/30	3.8	1.53	6.12
Mendiondo model MMSE 23/30 [13]	719	71.9	21/30	2	1.52	6.08
Zuin [31]	616	75	27/30	7.9	1.5	6.0
Stanley [43]	176	80.5	23.3/30	> 1	≥ 75 years: 1.3	5.2
Hager [24]	1021	73	19/30	2	1.07	4.28

Values are reported in decreasing order

**Fig. 1:**
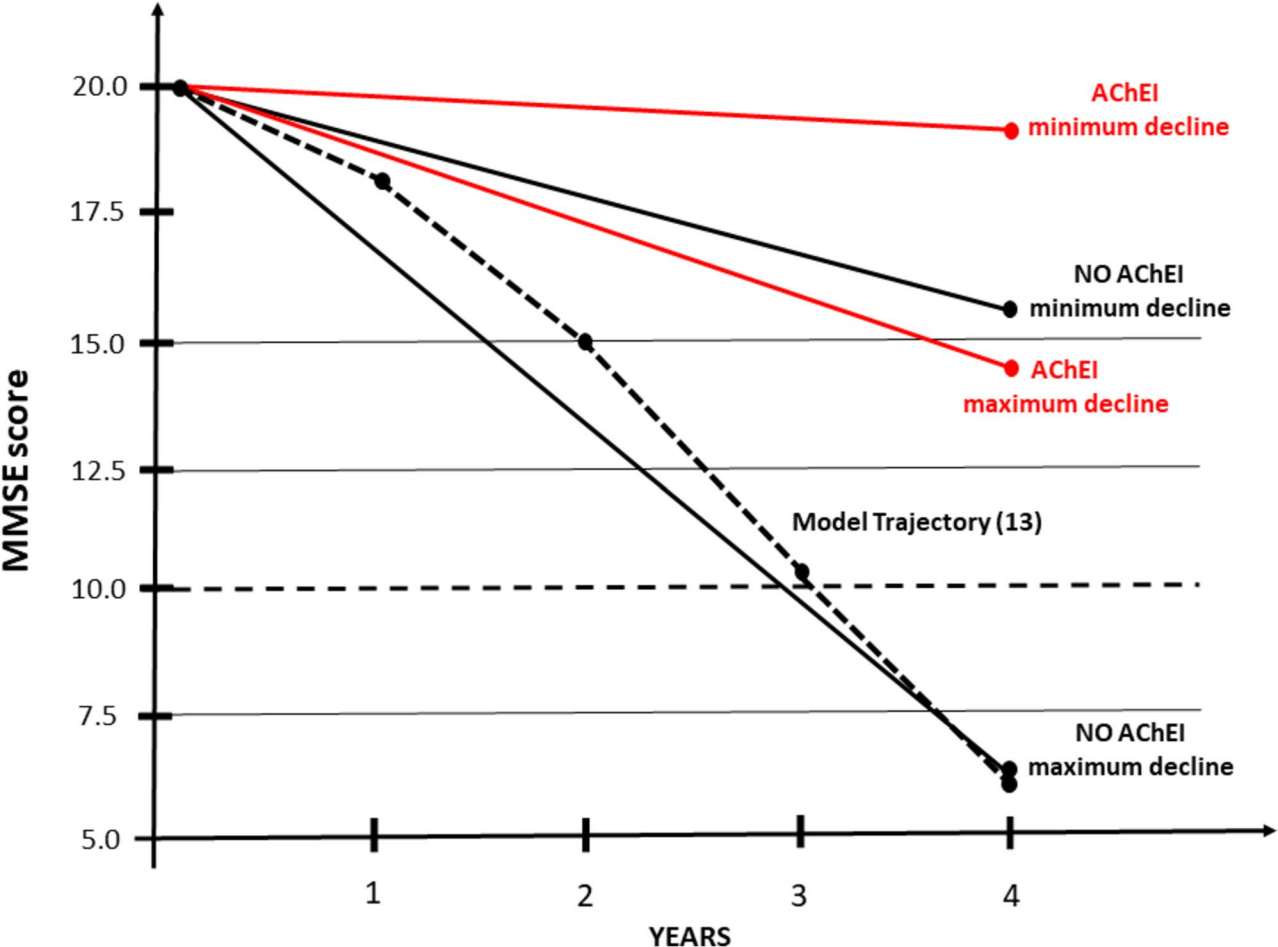
minimum and maximum mean loss in MMSE score in patients with AD treated or not-treated with AChEI, in a period of 4 years, as derived from the studies reported in the present study. The mean MMSE loss as calculated by the model of Mendiondo et al. [9] is also reported (dashed line)

In Fig. 1 are represented the range of MMSE decline in AD patients treated and not-treated with AChEI, in a period of 4 years, as derived from the studies reported in the present study, and from the model by Mendiondo [13].

## Discussion

### Effects of AChEI treatment

Although AChEI remains the most used drugs in the AD treatment, their real efficacy has been questioned. This might depend on three different factors. First, it is quite uncommon to notice a significant improvement in cognitive performance after starting AChEI treatment. Second, the AChEIs effects are “mild” and mainly consists of a slowdown/stabilization of cognitive decline. Finally, an important heterogeneity exists regarding the rate of AD progression. This last aspect reduces the possibility of understanding the real effect of the drug on the single patient. Nowadays, the general feeling is that, in the face of legitimate expectations on part of AD patients and their caregivers, AChEI have acquired, a little at a time, the reputation as an “ineffective” class of drugs. Theoretically, the “ideal” drug for AD would improve the cognitive functions and stop the disease progression. Unfortunately, the pharmaceutical research failed to find such a medication; this is probably due to the complexity of AD pathophysiology, which has not yet been fully understood, and to the potential irreversibility of the brain damage in AD. Thus, while the goal of reversing cognitive decline would be difficult to reach, the idea of slowing/stopping the progression would be a very acceptable goal.

With this premise, we evaluated the possible effect of AChEI on the rate of cognitive decline measured by MMSE, in AD patients from available “real-world” studies (open trials, databases, retrospective studies, and a few post-marketing RTCs). Indeed, phase-3 RCTs, besides having a short duration, enrol much-selected patients for well-known reasons; thus, they cannot provide evidence on the “real” efficacy of AChEI, especially over the long term. We found that, although partially overlapping, the rate of decline is slower in patients treated with AChEI compared with those not treated. The average loss of MMSE is in the order of 0.2–1.4 points/year in AD patients treated with AChEI, compared to 1.07–3.4 points/year in patients untreated. Note how the latest data is quite similar to those reported by Mendiondo in patients with mild-moderate AD from CERAD [13]. We are aware that, as pointed out by both Clark and Mendiondo [13, 37], there are some critical issue when using MMSE to measure the rate of cognitive decline. Indeed, a significant heterogeneity in the rate of change was reported, strongly depending on heterogeneity of the disease itself, but also on measurement error and inter-rater comparisons. However, although caution should be used when interpreting data obtained from MMSE, it could provide an “approximate” measure for cognitive change in AD patients over time, especially in case of longer follow-up.

Another interesting observation emerging from six studies included in our review is that AChEI treatment was associated with a 27–42% reduction in total mortality. Similar data have been reported in the meta-analysis by Truong et al. in patients affected by different types of dementia, including AD [45]. These authors reported a 23% reduction in all-cause mortality (adjusted HR: 0.77; 95%CI: 0.70–0.84) with no difference between the type of dementia, age, or individual drug. Several mechanisms have been proposed to explain this finding including: (1) Reduction in cardiovascular mortality, related to the reduction of peripheral cytokine and increase vagal nerve activity [23]; (2) Better compliance of patients treated with AChEI in the management of life style and medical problems; (3) Reduction of behavioral and psychological symptoms of dementia (BPSD), with secondary reduction of antipsychotic use and related mortality; (4) Slowdown of frailty progression related to the slowdown of cognitive decline; (5) “Confounding by indication” bias, since healthier subjects are more often prescribed AChEI.

### Comparison with other chronic degenerative diseases

Overall, it appears that, although AChEI do not stop the progression of AD nor ameliorate noticeably the cognitive functions, they might instead slowdown the progression of cognitive decline and contribute to reduce overall mortality in the long-term period.

The treatment of the most frequent chronic diseases, such as congestive heart failure (CHF), chronic obstructive pulmonary disease (COPD), and chronic kidney disease (CKD) has much improved in the last 20 years. Two are the major goals of these therapies: (1) Reducing the symptoms and the frequency of the exacerbations; (2) Slowing the progression of the disease thus delaying disability onset and increasing survival. Indeed, there is no etiological cure for chronic degenerative diseases; of consequence, the target of therapy is, paradoxically, to prolong the duration of the disease thereby prolonging the life of the patient.

In this context, we would like to compare the order of magnitude of the effect of drugs commonly used in CHF, COPD, and CKD with the effect of AChEI. In CHF with reduced ejection fraction (HFrEF), ACE-Inhibitors reduced mortality by 8–16%, while β-blockers reduced mortality by 31–34%, according to the MERIT-HF, CIBIC II and COPERNICUS trials [46]. In patients with CHF and atrial fibrillation (AF), β-blockers reduced mortality by 27%. Furthermore, in COPD, the inhalatory dual therapy reduced mortality by 12–17% compared to placebo or mono-therapy, while triple therapy further reduced mortality by 29–50% compared with dual therapy [47]. Similarly, in CKD, the treatment with ACE-Inhibitors reduced the risk of progression to end-stage disease by about 30% [48], while glifozines reduced by 30% the primary outcome (CKD progression or cardiovascular death) [49].

As regards the possible effect of AChEI in AD patients, we have brought some evidence to suggest that their use is associated with: (1) A slight/moderate slowdown in the progression of cognitive impairment (Fig. 1) [18, 21, 24, 31–33]; (2) A reduction in overall mortality, of the order of 25–35% [23–25, 28, 30, 31, 45]; Moreover, a slight reduction in the incidence of BPSD has been reported in Literature [50–52]. Basically, the clinical effects obtained with AChEI are of the same order of magnitude as the drugs used in the most common chronic disorders. Unfortunately, most of these data were obtained from open/non randomized or retrospective studies, and this may represent a problem since they might introduce several bias, unlike RCTs. However, as underlined by Birks and Raina [8, 53], the most important limit of the RCTs with AChEI included into their meta-analyses was the short term, most of the time comprised between 6 and 12 months. Of consequence, open and retrospective studies represent the best available evidence on the possible effect of AChEI in the context of the real-word.

## References

[1] Prince M, Bryce R, Albanese E et al (2013) The global prevalence of dementia: a systematic review and metaanalysis. Alzheimers Dement 9:63–75.e2.10.1016/j.jalz.2012.11.007 10.1016/j.jalz.2012.11.007 23305823

[2] Li X, Feng X, Sun X (2022). Global, regional, and national burden of Alzheimer’s disease and other dementias, 1990–2019. Front Aging Neurosci.

[3] Cervellati C, Trentini A, Pecorelli A (2020). Inflammation in neurological disorders: the thin boundary between brain and periphery. Antioxid Redox Signal.

[4] Rabinovici GD (2019). Late-onset Alzheimer disease. Contin Lifelong Learn Neurol.

[5] Cervellati C, Trentini A, Rosta V (2020). Serum beta-secretase 1 (BACE1) activity as candidate biomarker for late-onset Alzheimer’s disease. GeroScience.

[6] Budd Haeberlein S, Aisen PS, Barkhof F (2022). Two randomized phase 3 studies of aducanumab in early Alzheimer’s disease. J Prev Alzheimer’s Dis.

[7] van Dyck CH, Swanson CJ, Aisen P (2023). Lecanemab in early Alzheimer’s disease. N Engl J Med.

[8] Birks JS (2006). Cholinesterase inhibitors for Alzheimer’s disease. Cochrane Database Syst Rev.

[9] Marucci G, Buccioni M, Ben DD (2021). Efficacy of acetylcholinesterase inhibitors in Alzheimer’s disease. Neuropharmacology.

[10] Kobayashi H, Ohnishi T, Nakagawa R (2016). The comparative efficacy and safety of cholinesterase inhibitors in patients with mild-to-moderate Alzheimer’s disease: a Bayesian network meta-analysis. Int J Geriatr Psychiatry.

[11] Knight R, Khondoker M, Magill N (2018). A systematic review and meta-analysis of the effectiveness of acetylcholinesterase inhibitors and memantine in treating the cognitive symptoms of dementia. Dement Geriatr Cogn Disord.

[12] Dou K-X, Tan M-S, Tan C-C (2018). Comparative safety and effectiveness of cholinesterase inhibitors and memantine for Alzheimer’s disease: a network meta-analysis of 41 randomized controlled trials. Alzheimers Res Ther.

[13] Mendiondo MS, Ashford JW, Kryscio RJ (2000). Modelling mini mental state examination changes in Alzheimer’s disease. Stat Med.

[14] Small G, Kaufer K, Mendiondo MS (2005). Cognitive performance in Alzheimer’s disease patients receiving rivastigmine for up to 5 years. Int J Clin Pract.

[15] Bullock R, Dengiz A (2005). Cognitive performance in patients with Alzheimer’s disease receiving cholinesterase inhibitors for up to 5 years. Int J Clin Pract.

[16] Bellelli G, Lucchi E, Minicuci N (2005). Results of a multi-level therapeutic approach for Alzheimer’s disease subjects in the “real world” (CRONOS project): a 36-week follow-up study. Aging Clin Exp Res.

[17] Wallin AK, Andreasen N, Eriksson S (2007). Donepezil in Alzheimer’s disease: what to expect after 3 years of treatment in a routine clinical setting. Dement Geriatr Cogn Disord.

[18] Nelson PT, Kryscio RJ, Abner EL (2009). Acetylcholinesterase inhibitor treatment is associated with relatively slow cognitive decline in patients with Alzheimer’s disease and AD + DLB. J Alzheimers Dis.

[19] Calabria M, Geroldi C, Lussignoli G (2009). Efficacy of acetyl-cholinesterase-inhibitor (ACHEI) treatment in Alzheimer’s disease: a 21-month follow-up “real world” study. Arch Gerontol Geriatr.

[20] Wattmo C, Wallin AK, Londos E, Minthon L (2011). Predictors of long-term cognitive outcome in Alzheimer’s disease. Alzheimers Res Ther.

[21] Kavanagh S, Van Baelen B, Schäuble B (2011). Long-term effects of galantamine on cognitive function in Alzheimer’s disease: a large-scale international retrospective study. J Alzheimers Dis.

[22] Wallin AK, Wattmo C, Minthon L (2011). Galantamine treatment in Alzheimer’s disease: response and long-term outcome in a routine clinical setting. Neuropsychiatr Dis Treat.

[23] Nordström P, Religa D, Wimo A (2013). The use of cholinesterase inhibitors and the risk of myocardial infarction and death: a nationwide cohort study in subjects with Alzheimer’s disease. Eur Heart J.

[24] Hager K, Baseman AS, Nye JS (2014). Effects of galantamine in a 2-year, randomized, placebo-controlled study in Alzheimer’s disease. Neuropsychiatr Dis Treat.

[25] Wattmo C, Londos E, Minthon L (2015). Longitudinal associations between survival in Alzheimer’s disease and cholinesterase inhibitor use, progression, and community-based services. Dement Geriatr Cogn Disord.

[26] Nakano Y, Matsuzono K, Yamashita T (2015). Long-term efficacy of galantamine in Alzheimer’s disease: the Okayama Galantamine Study (OGS). J Alzheimers Dis.

[27] Nakagawa R, Ohnishi T, Kobayashi H (2017). Long-term effect of galantamine on cognitive function in patients with Alzheimer’s disease versus a simulated disease trajectory: an observational study in the clinical setting. Neuropsychiatr Dis Treat.

[28] Mueller C, Perera G, Hayes RD (2018). Associations of acetylcholinesterase inhibitor treatment with reduced mortality in Alzheimer’s disease: a retrospective survival analysis. Age Ageing.

[29] Vaci N, Koychev I, Kim C-H (2021). Real-world effectiveness, its predictors and onset of action of cholinesterase inhibitors and memantine in dementia: retrospective health record study. Br J Psychiatry.

[30] Xu H, Garcia-Ptacek S, Jönsson L (2021). Long-term effects of cholinesterase inhibitors on cognitive decline and mortality. Neurology.

[31] Zuin M, Cherubini A, Volpato S (2022). Acetyl-cholinesterase-inhibitors slow cognitive decline and decrease overall mortality in older patients with dementia. Sci Rep.

[32] Zurlo A (2005) Personal communication. Congresso regionale SIGG Bologna, Italy

[33] Bonati P (2005) Personal communication. Congresso regionale SIGG Bologna, Italy

[34] Corey-Bloom J, Galasko D, Hofstetter CR (1993). Clinical features distinguishing large cohorts with possible AD, probable AD, and mixed dementia. J Am Geriatr Soc.

[35] Rasmusson DX, Carson KA, Brookmeyer R (1996). Predicting rate of cognitive decline in probable Alzheimer’s disease. Brain Cogn.

[36] Agüero-Torres H, Fratiglioni L, Guo Z (1998). Prognostic factors in very old demented adults: a seven-year follow-up from a population-based survey in Stockholm. J Am Geriatr Soc.

[37] Clark CM, Sheppard L, Fillenbaum GG (1999). Variability in annual mini-mental state examination score in patients with probable Alzheimer disease: a clinical perspective of data from the Consortium to Establish a Registry for Alzheimer’s Disease. Arch Neurol.

[38] Han L, Cole M, Bellavance F (2000). Tracking cognitive decline in Alzheimer’s disease using the mini-mental state examination: a meta-analysis. Int Psychogeriatr.

[39] Doody RS, Massman P, Dunn JK (2001). A method for estimating progression rates in Alzheimer disease. Arch Neurol.

[40] Suh G-H, Ju Y-S, Yeon BK (2004). A longitudinal study of Alzheimer’s disease: rates of cognitive and functional decline. Int J Geriatr Psychiatry.

[41] Holmes C (2005). Rate of progression of cognitive decline in Alzheimer’s disease: effect of butyrylcholinesterase K gene variation. J Neurol Neurosurg Psychiatry.

[42] Tschanz JT, Corcoran CD, Schwartz S (2011). Progression of cognitive, functional, and neuropsychiatric symptom domains in a population cohort with Alzheimer dementia: the Cache County Dementia Progression Study. Am J Geriatr Psychiatry.

[43] Stanley K, Whitfield T, Kuchenbaecker K (2019). Rate of cognitive decline in Alzheimer’s disease stratified by age. J Alzheimer’s Dis.

[44] Jia J, Gauthier S, Pallotta S (2017). Consensus-based recommendations for the management of rapid cognitive decline due to Alzheimer’s disease. Alzheimer’s Dement.

[45] Truong C, Recto C, Lafont C (2022). Effect of cholinesterase inhibitors on mortality in patients with dementia: a systematic review of randomized and nonrandomized trials. Neurology.

[46] Xu T, Huang Y, Zhou H (2019). β-blockers and risk of all-cause mortality in patients with chronic heart failure and atrial fibrillation—a meta-analysis. BMC Cardiovasc Disord.

[47] Mintz M, Barjaktarevic I, Mahler DA (2023). Reducing the risk of mortality in chronic obstructive pulmonary disease with pharmacotherapy: a narrative review. Mayo Clin Proc.

[48] Jafar TH (2001). Angiotensin-converting enzyme inhibitors and progression of nondiabetic renal disease. Ann Intern Med.

[49] Herrington WG, Staplin N, Wanner C (2023). Empagliflozin in patients with chronic kidney disease. N Engl J Med.

[50] Cummings JL, Mega M, Gray K (1994). The Neuropsychiatric Inventory: comprehensive assessment of psychopathology in dementia. Neurology.

[51] Mega MS, Masterman DM, O’Connor SM (1999). The spectrum of behavioral responses to cholinesterase inhibitor therapy in Alzheimer disease. Arch Neurol.

[52] Birks JS, Harvey RJ (2018). Donepezil for dementia due to Alzheimer’s disease. Cochrane Database Syst Rev.

[53] Raina P, Santaguida P, Ismaila A (2008). Effectiveness of cholinesterase inhibitors and memantine for treating dementia: evidence review for a clinical practice guideline. Ann Intern Med.

